# Early improvement of daily physical activity after catheter ablation for atrial fibrillation in an accelerometer assessment: A prospective pilot study

**DOI:** 10.1111/anec.12807

**Published:** 2020-09-19

**Authors:** Satoshi Yanagisawa, Yasuya Inden, Aya Fujii, Yusuke Sakamoto, Toshiro Tomomatsu, Keita Mamiya, Hiroya Okamoto, Toyoaki Murohara, Rei Shibata

**Affiliations:** ^1^ Department of Advanced Cardiovascular Therapeutics Nagoya University Graduate School of Medicine Nagoya Japan; ^2^ Department of Cardiology Nagoya University Graduate School of Medicine Nagoya Japan

**Keywords:** accelerometer, atrial fibrillation, international physical activity questionnaire, physical activity, catheter ablation

## Abstract

**Background:**

Catheter ablation improves physical activity in patients with atrial fibrillation (AF). However, continuous daily evaluation and time course of improvement in physical activity after ablation have not been fully assessed. This prospective study was conducted to evaluate the daily physical activities and changes in the physical performance in patients undergoing catheter ablation for AF by continuous monitoring of a portable accelerometer.

**Methods:**

Ten patients scheduled for catheter ablation for AF were fitted with a uniaxial accelerometer prior to and 6 months after the procedure. This study evaluated changes in daily steps, activity intensity, and activity duration. We also evaluated changes in activity intensity using a short version of the International Physical Activity Questionnaire (IPAQ).

**Results:**

The maximum daily steps significantly increased from baseline to postablation (baseline, 9,232 [6,716–11,485]; after 1–3 months, 11,605 [8,285–14,802]; and after 4–6 months, 11,412 [8,939–13,808], *p* = .020). Similarly, Δ maximum‐mean daily steps increased significantly (baseline, 2,431 [1,199–6,181]; after 1–3 months, 4,674 [4,164–6,474]; and after 4–6 months, 4,871 [3,657–6,117], *p* = .014). These improvements were more pronounced in patients with paroxysmal and symptomatic AF. The total IPAQ score significantly improved from baseline to after 6 months ablation (from 1,170 [693–3,930] to 4,312 [1,865–6,569], *p* = .037). All patients were recurrence‐free from AF after ablation.

**Conclusions:**

The physical activity improved significantly even in the early phase following catheter ablation. The effect of suppressing AF on activity levels was apparent soon after the procedure.

## 1 INTRODUCTION

Atrial fibrillation (AF) is the most common arrhythmia in the present day and is frequently complicated with lethal cardiovascular diseases (Kannel et al., 1998; Wang et al., 2003). Symptomatic AF often presents as palpitation, dyspnea, and stagger, while leg edema and shortening of breath are present if the patients develop heart failure due to AF (Nabauer et al., 2009). These unfavorable symptoms can restrict physical activity and quality of life in the patients (Dorian et al., 2000). Patients with AF exhibited decreased oxygen uptake and exercise tolerance in a cardiopulmonary exercise test and treadmill test than in patients with sinus rhythm (Singh et al., 2006; Takano et al., 2019). Excessive increase in heart rate response to exercise, hemodynamical impairment of rhythm regularity, and loss of atrial contraction could be possible explanations for the lower level of physical activity in patients with AF.

Catheter ablation is an acceptable therapeutic approach for suppressing AF with adequate successful results in clinical practice. Although restoring sinus rhythm by pharmacological approach and electrical cardioversion was reported to improve exercise capacity, catheter ablation could also improve exercise tolerance and quality of life with better maintenance of sinus rhythm as compared to the former approaches in previous reports (Fiala et al., 2014; Haissaguerre et al., 2005; Jones et al., 2013; Katayama et al., 2019; Katsumata et al., 2019; Mohanty et al., 2014; Ueshima et al., 1993; Yagishita et al., 2017). Generally, the exercise capacity and tolerance are evaluated by means of cardiopulmonary exercise or treadmill tests at the time of each follow‐up period. However, these evaluations were intermittent and limited by a fixed time. Unstable conditions and abnormal rhythm at the time of evaluation could affect the exercise performance. Moreover, continuous daily evaluation and time course of recovery of physical activity after catheter ablation has not been fully assessed. It is unclear when and how the impaired physical activity recovers by restoring sinus rhythm after the catheter ablation.

The present study was conducted to evaluate daily physical activity in patients undergoing catheter ablation for AF by using a portable accelerometer. We examined the extent of recovery of physical activity and changes in the physical performance after the procedure by continuous monitoring of an accelerometer.

## 2 METHODS

### 2.1 Study population

This prospective study included patients who were scheduled for catheter ablation for AF from June 2016 to March 2019 in Nagoya University Hospital, Japan. All the patients agreed and gave written informed consent for the present study before enrollment. The indications for catheter ablation were in compliance with the latest guidelines (Calkins et al., 2017). The patients were offered to be fitted with the accelerometer prior to and for 6 months of follow‐up after the procedure. A total of 10 patients with adequate data sample and follow‐up were analyzed for the present study. Antiarrhythmic agents were discontinued at least 5 drug half‐lives before the ablation. Prior to the procedure, informed consent was obtained from all patients. This study was approved by our institutional ethics committee. This trial is registered with the UMIN Clinical Trials Registry numbered: UMIN000025979. The study was performed in compliance with the principles of the Declaration of Helsinki.

### 2.2 Catheter ablation procedures

The patients were administered oral anticoagulants at least 3–4 weeks before the ablation procedure. All the patients were admitted the day before the procedure. At admission, baseline blood tests, echocardiography, and electrocardiography were performed. All the patients underwent a transesophageal echocardiography before the procedure to confirm the absence of an atrial thrombus.

Vascular access was obtained via the right femoral and left subclavian veins. This study included two catheter ablation techniques: radiofrequency and cryoballoon ablations. In the radiofrequency ablation procedure, after the administration of a heparin bolus, two 8‐Fr sheaths and an 8.5‐Fr steerable sheath were introduced into the left atrium through a trans‐septal puncture using intracardiac echocardiography. Using a circular mapping catheter placed on the ostium of the pulmonary vein (PV), encircling PV isolation was performed with a 3.5‐mm tip, open‐irrigated contact force‐sensing ablation catheter. All ablation procedures were performed using a three‐dimensional mapping system. The radiofrequency energy output was adjusted to 25–35 W at a flow rate of 8–17 ml/min with a maximum temperature of 42°C. In most patients with paroxysmal AF and early persistent AF, we performed PV isolation alone. However, for the patients with atrial flutter, atrial tachycardia, or long‐term persistent AF, additional ablations including linear ablation, complex fractionated atrial electrogram ablation, and substrate ablation were applied. If the rhythm did not convert to sinus rhythm at the end of the ablation, external cardioversion was performed.

For the cryoballoon ablation procedure, a 12‐Fr steerable sheath was introduced into the left atrium. A second‐generation 28‐mm cryoballoon system (Arctic Front Advance, Medtronic) was advanced and placed on the ostium of each PV using an inner circular mapping catheter. After confirmation of the PV ostium occlusion with the cryoballoon using the contrast medium, a 120–180 s cycle freeze ablation was repeated until electrical isolation of the PV was achieved.

During the entire procedure, the activated clotting time was monitored every 20–30 min after the bolus infusion. The target range for an activated clotting time between 300 and 350 s was maintained by additional heparin infusion. After the ablation procedure, patients were followed under continuous electrocardiogram monitoring at the hospital for 3 days and then discharged.

### 2.3 Follow‐up

All patients were followed‐up at the outpatient clinic in our hospital. At each follow‐up visit for a minimum duration of 1 month, 3‐min electrocardiography was examined, and any symptom related to arrhythmia was considered. Twenty‐four‐hour Holter monitoring examination was performed in all patients one month after the ablation. If recurrence was suspected, additional short‐term follow‐up visit and repeat Holter monitoring test was arranged to detect the recurrence as much as possible. Recurrence was defined as any AF and atrial tachycardia lasting ≥30 s detected on examination after a blanking period of 3 months after the procedure.

### 2.4 Accelerometer assessment for the daily activity

All patients were fitted an activity monitor with a uniaxial accelerometer (Kenz Lifecorder GS; Suzuken Co, Ltd) during the study period. This device is utilized by attaching it to a person's waist. After obtaining informed consent from the patient for the study enrollment before the ablation, we demonstrated how to use and fit the accelerometer by showing it to the patient. The baseline information of the patients’ weight and height, time, and date were input into the portable machine by the attending doctor manually. Since the accelerometer use is very simple, as it only has to be fitted on the waist after the initial set up, all the patients understood the method with 5–10 min guidance without needing further complex training at the beginning. The patients were advised to use the device during all waking hours, except for bathing, to live as routine a life as possible, and were encouraged to fit this accelerometer at least once per week. This device measured the daily step count and time of the physical activity intensity every day. The sensor of the accelerometer determined the extent of physical activity level with a 10‐grade evaluation (0–9). A correlation of the specific grade evaluation with metabolic equivalents (METs) has been confirmed by a previous study (Kumahara et al., 2004). Activity intensity levels were classified as light (1–3 activity grade), moderate (4–6 activity grade), and vigorous (7–9 activity grade) categories. The battery life used in this device could last for approximately 200 days; therefore, we could collect physical activity data for a long duration of ≥6 months during the follow‐up. At each regular follow‐up visit, we requested the patients for compliance of fitting the accelerometer during the follow‐up period. Additionally, we encouraged the patients to continue the fitting thereafter. The data recorded in this device were analyzed by a specific off‐line analyzer software (Lifelizer Coach 05; Suzuken Co, Ltd, Nagoya, Japan) after retrieving the accelerometer from the patient at the end of the study. An intermittent online assessment of the patient data during the study period was not systematically performed in this study.

We divided the total study duration into 3 terms: before ablation procedure (study inclusion to the procedure; 3–4 weeks), 1–3 months after ablation, and 4–6 months after ablation. Parameters evaluated in this study were the mean and maximum number of steps taken daily, Δ maximum‐mean daily steps, activity duration, intensity, and duration of the physical activity level at an intensity of activity grade level ≥4, during each follow‐up period.

### 2.5 Physical activity assessment with a questionnaire

The study also assessed the physical activity levels by a short version of International Physical Activity Questionnaire (IPAQ) prior to and 6 months after ablation. The short version of IPAQ form is an instrument designed primarily for population surveillance of physical activity among adults ("International Physical Activity Questionnaires IPAQ: Short Last 7 Days Self‐Administered Format http://www.sdp.univ.fvg.it/sites/default/files/IPAQ_English_self-admin_short.pdf (Accessed Dec 15, 2019)"). IPAQ assessment provides separate, specific scores for walking‐intensity, moderate‐intensity, and vigorous‐intensity activities by summation of the duration (in minutes) and frequency (days). The activity score was calculated by walking MET‐minutes/week = 3.3 × walking minutes × walking days; moderate MET‐minutes/week = 4.0 × moderate‐intensity activity minutes × moderate days; and vigorous MET‐minutes/week = 8.0 × vigorous‐intensity activity minutes × vigorous‐intensity days. The total physical activity MET‐minutes/week of the patient was sum of walking + moderate +vigorous MET‐minutes/week scores.

### 2.6 Statistical analysis

Continuous variables are expressed as mean ± standard division or median (first and third quartiles). Differences between the baseline and follow‐up in outcome parameters were compared using a paired *t* test or Wilcoxon rank sum test. To test for a significant difference in means over time for the same subjects, a repeated measures analysis of variance for parametric and Friedman test for a nonparametric were used. A *p*‐value of >.05 was considered as statistically significant.

## 3 RESULTS

### 3.1 Baseline patient characteristics and procedure results

Baseline characteristics and examination results are shown in Table 1. Mean age was 71.6 ± 7.8 years, and 60% of the patients were male. Half the patients had paroxysmal AF. Mean CHADS2 and CHA2DS2‐VASc scores were 1.5 ± 1.1 and 3.0 ± 1.5, respectively. Left ventricular ejection fraction < 40% on echocardiography was found in 2 patients. Seven patients underwent radiofrequency catheter ablation, while three patients underwent cryoballoon ablation. All patients underwent ablation procedure without any complication, except for one patient with complicating cardiac tamponade at the end of the ablation. However, the patient fully recovered after pericardiocentesis and was discharged 5 days later without an obvious decrease in physical activity.

**Table 1:** Baseline characteristics and examination results

	Total (*n* = 10)
Age, years	71.6 ± 7.8
Male sex	6 (60%)
Body weight, kg	61.5 ± 17.5
Body mass index, kg/m^2^	23.6 ± 3.9
Duration of AF, years	0.6 (0.3–1.6)
Paroxysmal AF	5 (50%)
Symptoms	6 (60%)
Comorbidities	
Hypertension	6 (60%)
Diabetes mellitus	2 (20%)
Heart failure	3 (30%)
Coronary artery disease	2 (20%)
Stroke	0 (0%)
Echocardiographic data	
Left atrial diameter, mm	47.7 ± 7.0
Left ventricular ejection fraction, %	55.1 ± 13.5
Left ventricular ejection fraction < 40%	2 (20%)
CHADS2 score	1.5 ± 1.1
CHA2DS2‐VASc score	3.0 ± 1.5
Ablation procedures	
Radiofrequency ablation	7 (70%)
Cryoballoon ablation	3 (30%)

Note

Data are expressed as mean ± standard division or median (first and third quartiles).

Abbreviation: AF, atrial fibrillation.

As for prognosis, two patients had an early recurrence within 1 and 2 days after the procedure, respectively. However, subsequent oral antiarrhythmic drugs could suppress the early recurrence immediately resulting in sinus rhythm, and they could maintain sinus rhythm by taking antiarrhythmic drugs thereafter. No patient had recurrence during the follow‐up period of 6 months after ablation. Two patients were administered class III antiarrhythmic drugs and one patient with class I antiarrhythmic drugs at the follow‐up period.

### 3.2 Changes in physical activity parameters in the accelerometer

**Figure 1:**
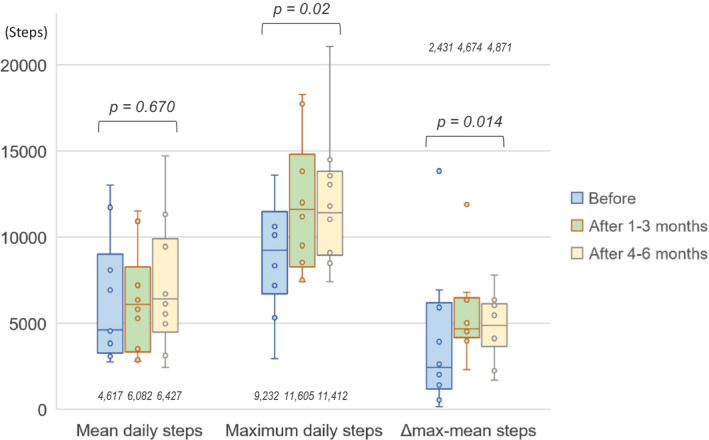
Changes in daily steps on the accelerometer from baseline to postablation. The values in the bar graph indicate median data

During the total study duration, the mean days the accelerometer was fitted was 111 ± 58 days. In the total population, the mean daily steps increased after the procedure (baseline, 4,617 [3,281–9,001], after 1–3 months, 6,082 [3,356–8,277], and after 4–6 months, 6,427 [4,510–9,919], respectively, *p* = .670). The maximum daily steps significantly increased from baseline to postablation (baseline, 9,232 [6,716–11,485], after 1–3 months, 11,605 [8,285–14,802], and after 4–6 months, 11,412 [8,939–13,808], respectively, *p* = .020). Similarly, Δ maximum‐mean daily steps increased significantly (baseline, 2,431 [1,199–6,181], after 1–3 months, 4,674 [4,164–6,474], and after 4–6 months, 4,871 [3,657–6,117], respectively, *p* = .014) (Figure 1 and Table 2).

**Table 2:** Changes in the physical activity parameters from baseline to postablation

Parameter	Before 3–4 weeks	After 1–3 months	After 4–6 months	*p*‐value
Mean daily steps	4,617 (3,281–9,001)	6,082 (3,356–8,277)	6,427 (4,510–9,919)	.670
Maximum daily steps	9,232 (6,716–11,485)	11,605 (8,285–14,802)	11,412 (8,939–13,808)	.020
Δ Maximum‐mean daily steps	2,431 (1,199–6,181)	4,674 (4,164–6,474)	4,871 (3,657–6,117)	.014
Total activity time/day, min	64.6 ± 38.6	66.9 ± 33.1	74.9 ± 40.1	.147
Light activity time/day, min	48.7 ± 28.7	50.8 ± 25.7	57.4 ± 31.0	.158
Moderate activity time/day, min	15.7 ± 14.9	15.7 ± 9.8	17.0 ± 12.0	.818
Vigorous activity time/day, min	0.2 ± 0.3	0.4 ± 0.5	0.5 ± 0.7	.152
Maximum activity time/day, min	99.8 (66.2–119.0)	119.6 (83.3–145.8)	112.1 (85.7–142.0)	.067
Maximum activity time (≥ moderate)/day, min	23.0 (11.8–31.9)	30.2 (18.6–50.9)	33.6 (17.2–56.3)	.061

Note

Data are expressed as mean ± standard division or median (first and third quartiles). Activity intensity levels in the accelerometer were classified for light (1–3 activity grade), moderate (4–6 activity grade), and vigorous (7–9 activity grade) categories.

The total activity time gradually increased after the procedure (baseline, 64.6 ± 38.6 min, after 1–3 months, 66.9 ± 33.1 min, and after 4–6 months, 74.9 ± 40.1 min, respectively, *p* = .147) (Table 2). In a detailed analysis, the light and vigorous activity times mainly improved after the procedure. There was a trend toward increased maximum activity time and maximum activity time (≥ moderate intensity) after the procedure (baseline, 99.8 [66.2–119.0] min, after 1–3 months, 119.6 [83.3–145.8] min, and after 4–6 months, 112.1 [85.7–142.0] min, *p* = .067; and baseline, 23.0 [11.8–31.9] min, after 1–3 months, 30.2 [18.6–50.9] min, and after 4–6 months, 33.6 [17.2–56.3] min, *p* = .061).

### 3.3 Changes in physical activity in the questionnaire assessment

**Figure 2:**
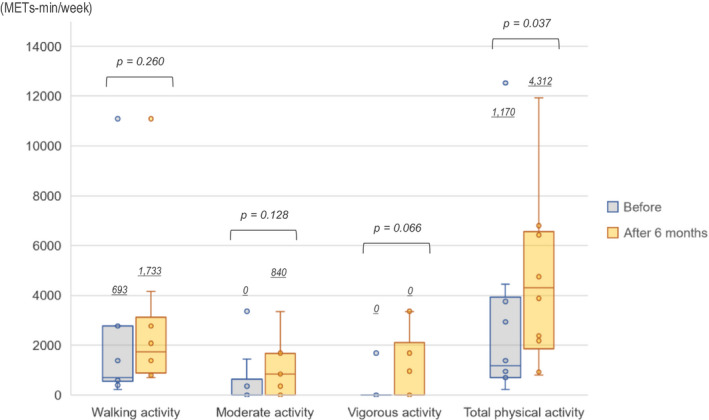
Changes in the IPAQ scores from baseline to 6 months after ablation. IPAQ, international physical activity questionnaire; METs, metabolic equivalents. The values in the bar graph indicate median data

Changes in the IPAQ scores from baseline to postablation are shown in Table 3 and Figure 2. Walking, moderate, and vigorous MET‐minutes/week were increased at the 6‐month follow‐up. The total physical activity MET‐minutes/week was significantly improved from baseline until 6 months after ablation (1,170 [693–3,930] to 4,312 [1,865–6,569], *p* = .037).

**Table 3:** Changes in the IPAQ scores from baseline to 6 months after ablation

Parameters	Before 3–4 weeks	After 6 months	*p*‐value
Walking MET‐minutes/week	693 (545–2,772)	1,733 (891–3,119)	.260
Moderate MET‐minutes/week	0 (0–630)	840 (0–1,680)	.128
Vigorous MET‐minutes/week	0 (0–0)	0 (0–2,100)	.066
Total physical activity MET‐minutes/week	1,170 (693–3,930)	4,312 (1,865–6,569)	.037

Note

Data are expressed as median (first and third quartiles). Walking MET‐minutes/week = 3.3 × walking minutes × walking days; moderate MET‐minutes/week = 4.0 × moderate‐intensity activity minutes × moderate days; vigorous MET‐minutes/week = 8.0 × vigorous‐intensity activity minutes × vigorous‐intensity days; Total physical activity MET‐minutes/week = sum of walking + moderate +vigorous MET‐minutes/week scores.

Abbreviations: IPAQ, international physical activity questionnaire; METs, metabolic equivalents.

### 3.4 Changes in physical activity parameters in subgroup analyses

The population was divided into the paroxysmal AF (*n* = 5) and persistent AF (*n* = 5) groups. The maximum daily steps and Δ maximum‐mean daily steps improved in parallel after the ablation in both groups (Supplemental Table S1).

## 4 DISCUSSION

This prospective pilot study evaluated continuous changes in physical activity after catheter ablation of AF using the portable accelerometer and IPAQ questionnaire assessments. Our major findings were as follows: (a) The maximum and Δ maximum‐mean daily steps significantly increased after the ablation, and improvement has been observed in the early phase after the ablation (b) The total activity time, especially light and vigorous activity time, increased after the procedure (c) The IPAQ score also significantly improved after the ablation.

Improved physical activity after the catheter ablation for persistent AF was reported in several studies. Maximal oxygen consumption and METs significantly increases after successful ablation at 6–12 months follow‐up in patients with long‐standing persistent AF, and even in asymptomatic patients (Fiala et al., 2014; Mohanty et al., 2014). The favorable physical parameters were also observed in cardiopulmonary exercise testing 3 months after ablation (Katayama et al., 2019; Katsumata et al., 2019). However, these assessments of physical activities adopted an intermitted evaluation of cardiopulmonary exercise test at the follow‐up visits. Further, follow‐up assessments were mostly 3–12 months after ablation at the earliest. One previous study showed a significant increase in exercise duration on bicycle ergometry from 1 to 6 months after ablation for AF in patients with sinus rhythm (Haissaguerre et al., 2005). However, in that study, exercise performance prior to ablation was not evaluated, and changes between before and after the procedures were not assessed. It remains unclear how and when the physical activity could recover after catheter ablation for AF. The present study is unique in evaluating changes in physical activity after the ablation through a continuous assessment by using a potable accelerometer. Surprisingly, the mean and maximum daily steps remarkably increased even at an early phase of 1–3 months after the ablation. Generally, soon after the ablation procedure, the pain from the puncture sites where catheter sheaths were inserted could be sometimes notable, which could restrict the daily physical activity. In addition, catheter ablation procedure to the left atrium affects the autonomic nervous system (Yanagisawa et al., 2018). An unbalanced condition between parasympathetic and sympathetic activity levels sometimes causes discomfort in the patients, and this could limit the daily activity. In this regard, our results showing an early recovery of physical activity after ablation suggest that these estimated adverse effects may be minimum and have little influence on the physical condition soon after procedure to the extent of limiting the activity level. The results would be helpful to establish a novel indicator for time needed to come back to their work and daily life after discharge of catheter ablation for AF. Moreover, our study showed that the remarkable changes were strongly observed in the maximum and Δ maximum‐mean daily steps rather than in the mean daily step after ablation. The result may indicate that maintenance of sinus rhythm after catheter ablation for AF may preferentially enlarge the capacity of activity levels at the time of exercise and workload, rather than the mean amount of activity duration at daily behavior.

Fortunately, all the patients in the present study did not have a recurrence at follow‐up, and adding antiarrhythmic drugs for early recurrence in 2 patients could prevent the AF burden thereafter. Restoring and maintenance of the sinus rhythm was essential to obtain the remarkable improvement of physical activity after ablation. Fiala et al. reported that the maximum oxygen consumption during exercise test was significantly increased in patients without recurrence at 6–12 months follow‐up after catheter ablation for persistent AF, in contrast, an improvement was not observed in patients with recurrence (Fiala et al., 2014). Other previous reports also demonstrated better recovery of the physical parameters after ablation while focusing on patients who were recurrence‐free (Katayama et al., 2019; Katsumata et al., 2019; Mohanty et al., 2014). AF itself is a reason for decreased physical activity. Increased heart rate at rest causing a reduction of stroke volume and loss of atrial function can be considered to be associated with decreased physical function in patients with AF (Pardaens et al., 1997; Takano et al., 2019). Furthermore, the uncomfortable symptoms related to AF due to irregularity or tachycardia rhythm may also limit their daily activity and physical function (Nabauer et al., 2009). Indeed, Proietti et al. demonstrated that daily activity decreased after daily burden of more than 500 min of AF by the assessment of insertable cardiac monitors in patients undergoing catheter ablation, although the exact time course of activity levels and its recovery after ablation was unknown in their study (Proietti et al., 2018). The present study was similar to the former studies with improved physical activity in all patients without late recurrence. Further studies would be needed to assess whether early improvement of physical activity could be observed in patients with early and late recurrences after ablation.

The present study could confirm the similar improvement of the activity level both in the questionnaire and accelerometer evaluations. Since the evaluation of the questionnaire completely depends upon the memory of the patient, there might be missing data regarding the previous recent activity level due to ambiguous memory of the patient. In contrast, a quantitative evaluation of the activity intensity using 10 grade levels and accurate activity time duration using an accelerometer has the merit to compare and assess objectively. It could be valuable to confirm a similar improvement of the activity both from the viewpoint of the patients and the objective point of view in the machine in this study.

### 4.1 Study limitations

This study was performed at a single institution, and the sample size was relatively small in a pilot study. Therefore, the outcomes before and after ablation may have not reached statistically significance owing to this small sample size. Although we made every effort and several examinations to detect recurrence after ablation at the follow‐up, the assessment of the recurrence detection after the ablation was not enough to detect a short duration of asymptomatic AF or atrial tachycardia, which may have underestimated the recurrence rate in this study. The accelerometer used in the present study was uniaxial. A multiple‐axial accelerometer could have detected acceleration in a more accurate manner and motion of the activity with a higher sensitivity than the uniaxial accelerometer. Moreover, as for the inclusion criteria, we included the patients who were likely to be interested in their own daily activity and health care management that could have resulted in better outcomes on physical parameters after ablation. In addition, there might be a possible placebo effect that cannot be determined without a control group. Further studies for evaluating the activity in patients with and without recurrence at the follow‐up in additional sample sized patients and assessing the true effect of AF ablation with comparison to the non‐ablation patients administered with pharmacological drugs intervention or non‐AF ablation group would be required.

## 5 CONCLUSIONS

The physical activity assessed by a portable accelerometer and questionnaire improved significantly after catheter ablation for AF. The effect of suppressing AF by successful catheter ablation on the activity levels was seen soon after the procedure. Our results could contribute to establishing a novel indicator of management and advise for daily physical activity and condition in patients undergoing catheter ablation for AF soon after discharge.

## CONFLICT OF INTEREST

Drs. Yanagisawa and Shibata are affiliated with a department sponsored by Medtronic Japan. Other authors have no conflict of interest.

## AUTHOR CONTRIBUTIONS

S.Y. designed the study, analyzed the data and wrote the manuscript. Y.I., A.F., Y.S., T.T., K.M., and H.O. helped the data interpretation. S.Y., Y. I., A.F., Y.S., T.T., K.M., and H.O. carried out the examination. Y.I. edited and commented the manuscript. T.M. and R.S. supervised this work. All authors reviewed the manuscript.

## ETHICAL APPROVAL

This study was approved by our institutional ethics committee. All the patients agreed and gave written informed consent for the present study before enrollment. The study was performed in compliance with the principles of the Declaration of Helsinki.

## Supporting information

Table S1

## Data Availability

The data that support the findings of this study are available from the corresponding author upon reasonable request.
