# Supplementary material for: Early improvement of daily physical activity after catheter ablation for atrial fibrillation in an accelerometer assessment: A prospective pilot study
Source: Ann Noninvasive Electrocardiol. 2020 Sep 19;26(1):e12807. doi: 10.1111/anec.12807 (PMC7816803; doi:10.1111/anec.12807)
Supplement: Supplementary file 1 — Table S1 [file ANEC-26-e12807-s001.doc]

Supplemental Table 1. Changes in the physical activity parameters from baseline to post-ablation in patients with paroxysmal and persistent AF

| *Parameters* | *Before* | *After 1–3 months* | *After 4–6 months* | *P-value* |
| --- | --- | --- | --- | --- |
| Mean daily steps |  |  |  |  |
| Paroxysmal AF | 4,549 (3,463–7,514) | 6,362 (2,828–7,299) | 6,714 (3,997–8,146) | 0.819 |
| Persistent AF | 4,685 (3,051–12,373) | 5,802 (4,405–11,220) | 6,139 (4,051–13,016) | 0.549 |
| Maximum daily steps |  |  |  |  |
| Paroxysmal AF | 8,342 (6,251–10,453) | 11,196 (7,454–15,138) | 11,024 (8,787–14,040) | 0.022 |
| Persistent AF | 10,610 (5,112–19,572) | 12,163 (9,028–15,781) | 11,799 (8,283–17,052) | 0.247 |
| Δ Maximum-mean daily steps |  |  |  |  |
| Paroxysmal AF | 2,229 (1,719–4,790) | 4,620 (4,258–8,314) | 5,474 (3,188–6,914) | 0.022 |
| Persistent AF | 3,937 (374–9,879) | 5,026 (3,265–6,588) | 4,268 (2,958–6,006) | 0.091 |
| Total activity time/day, min |  |  |  |  |
| Paroxysmal AF | 56.8 ± 23.2 | 57.6 ± 27.0 | 65.8 ± 27.1 | 0.392 |
| Persistent AF | 72.5 ± 51.5 | 76.3 ± 39.0 | 83.9 ± 51.8 | 0.434 |
| Light activity time/day, min |  |  |  |  |
| Paroxysmal AF | 46.4 ± 18.2 | 45.1 ± 22.7 | 51.5 ± 21.9 | 0.392 |
| Persistent AF | 51.0 ± 38.8 | 56.5 ± 29.8 | 63.3 ± 39.9 | 0.359 |
| Moderate activity time/day, min |  |  |  |  |
| Paroxysmal AF | 10.3 ± 7.2 | 12.1 ± 8.2 | 13.8 ± 9.6 | 0.567 |
| Persistent AF | 21.2 ± 19.3 | 19.3 ± 10.9 | 20.1 ± 14.3 | 0.868 |
| Vigorous activity time/day, min |  |  |  |  |
| Paroxysmal AF | 0.1 ± 0.1 | 0.4 ± 0.5 | 0.5 ± 0.7 | 0.313 |
| Persistent AF | 0.3 ± 0.5 | 0.5 ± 0.5 | 0.5 ± 0.8 | 0.454 |
| Maximum activity time/day, min |  |  |  |  |
| Paroxysmal AF | 87.1 ± 22.0 | 116.6 ± 39.9 | 115.6 ± 22.6 | 0.035 |
| Persistent AF | 124.5 ± 73.2 | 125.0 ± 40.0 | 125.2 ± 52.9 | 0.998 |
| Maximum activity time (≥ moderate)/day, min |  |  |  |  |
| Paroxysmal AF | 19.8 ± 14.1 | 36.3 ± 37.2 | 35.5 ± 22.1 | 0.208 |
| Persistent AF | 47.3 ± 55.3 | 37.6 ± 12.7 | 42.4 ± 29.4 | 0.803 |

AF, atrial fibrillation.
